# Prevalence and risk factors of knee osteoarthritis: a cross-sectional survey in Nanjing, China

**DOI:** 10.3389/fpubh.2024.1441408

**Published:** 2024-11-13

**Authors:** Wenjuan Shao, Huisheng Hou, Qi Han, Keshu Cai

**Affiliations:** ^1^College of Physical Education, Minzu University of China, Beijing, China; ^2^Sports Nutrition Center, National Institute of Sports Medicine, Beijing, China; ^3^Rehabilitation Medicine Center, The First Affiliated Hospital of Nanjing Medical University, Nanjing, China; ^4^Department of Rehabilitation, Nanjing Qixia District Hospital, Nanjing, China

**Keywords:** knee osteoarthritis, prevalence, risk factors, middle aged and older adult, China

## Abstract

**Background:**

Knee osteoarthritis (KOA) presents a significant public health challenge due to its hazards and increasingly severe trends. Addressing this challenge requires targeted investigation into the prevalence and identification of risk factors for KOA across different regions, especially in populous and vast China. Therefore, a cross-sectional survey was conducted in Nanjing, China, with the aim of investigating the prevalence and risk factors of KOA among individuals aged 50 and above.

**Method:**

A total of 1,045 subjects were selected using the stratified random sampling method and diagnosed with KOA based on the diagnostic criteria established by the Chinese Medical Association. Data on 14 potential risk factors were collected through a self-designed questionnaire and standardized on-site tests. The association between KOA and these risk factors was explored using *t*-tests, Chi-square tests, and logistic regression analysis.

**Results:**

The prevalence of KOA among the subjects was 23.64%. Multiple logistic regression models indicated that the risk of KOA was significantly higher among women (OR: 5.34, 95% CI: 3.13–9.11), subjects aged 60–69 (OR: 1.83, 95% CI: 1.25–2.69) and over 70 (OR: 2.87, 95% CI: 1.80–4.59), individuals with high school education and above (OR:2.22, 95% CI: 1.37–3.60), those with flatfoot (OR: 1.74, 95% CI: 1.10–2.74), and subjects classified as overweight (OR: 1.91, 95% CI: 1.21–3.04) and obese (OR: 4.63, 95% CI: 2.18–9.85) based on their BMI status. Additionally, the models identified weight (OR: 1.04, 95% CI: 1.01–1.08), 30-s chair stand performance (OR: 0.94, 95% CI: 0.91–0.97), and single-leg stand performance (OR: 0.96, 95% CI: 0.93–0.99) as independent risk factors for KOA.

**Conclusion:**

The prevalence of KOA is remarkable in Nanjing city. The risk factors for KOA include women, older age, higher education, flatfoot, increased weight and BMI, as well as poor performance in 30-s chair stand and single-leg stand tests.

## 1 Introduction

Knee osteoarthritis (KOA) is a chronic and irreversible joint disease accompanied by pain and limited joint activity (1, 2). It may pose difficulties for patients in their daily activities such as bathing, toileting, dressing, walking, and household chores (3). It may also lead to psychological problems such as insomnia, anxiety, and depression due to long-term pain and inconvenience (1). In severe cases, KOA may even cause patients to lose their ability of live independently and experience disabilities (4, 5). Evidence suggests that KOA is the fourth leading cause of disability for women and the eighth leading cause for men (6). Its disability rate ranks high among all disabling diseases worldwide (5, 7). Meanwhile, almost all epidemiological surveys indicate a continued increase in the prevalence of KOA in recent decades (7–9). According to the 2019 Global Burden of Disease Study, approximately 364.58 million people were suffering from KOA, with an age-standardized prevalence of 4.38%, and the estimated annual percentage growth was 0.32% from 1990 to 2019 (7). Given these hazards and prevalence trends, KOA has become a major public health challenge.

Investigating the prevalence and risk factors of KOA is a prerequisite for addressing this challenge, but its findings cannot be easily generalized. Studies have indicated that the prevalence of KOA among individuals aged 60 and above in the United States was 37.4%, whereas in Japan and Germany, it stood at 26.1 and 12.3%, respectively (10–12). A systematic review indicated that the prevalence of KOA in China was 21.5% (13), while the China Health and Retirement Longitudinal Study suggested a lower rate of 8.1% (14). These discrepancies in KOA prevalence may be attributed to various social risk factors, including population dynamics, economic development, and geographical location (9, 11). For example, the prevalence in rural areas of the United States was significantly higher than that in urban areas due to the impact of economic development (10), and the prevalence in France decreased gradually from northeast mountainous areas to southwest coastal plain due to the impact of geographical location (15). Moreover, evidences also suggest that personal characteristics and lifestyle factors such as gender, education, obesity, aging, smoking, drinking, and prolonged sitting may all be associated with KOA prevalence, but the effects of these factors vary accross different studies (1, 16–19). For instance, Ji Shuqing et al. reported that the risk of KOA was 1.51 and 2.24 times higher in overweight and obese individuals, respectively, compared to normal-weight individuals (20), while Ren Yan et al. found no significant differences of KOA risk among overweight, obese and normal-weight individuals (16). These discrepancies highlight the diversity and uncertainty of KOA risk factors, emphasizing the necessity of targeted identification, especially in a vast and populous country like China.

This study was carried out in Nanjing, a city in southern China. China has conducted fewer cross-sectional surveys on KOA compared to developed countries (14). Simultaneously, Nanjing has barely reported a KOA survey in the past 10 years, according to our systematic search on Web of Science and PubMed databases. In order to enrich the epidemiological data on KOA, investigate the current prevalence and risk factors of KOA, and develop targeted strategies for KOA prevention and control in Nanjing, we designed and conducted this study.

## 2 Subjects and methods

This study obtained ethical approval from Nanjing Qixia District Hospital (No. 2022QX0901). It was conducted by standardized trained general practitioners and nursing staff at two community hospitals in Qixia District from September to October 2022.

### 2.1 Subjects

The random stratified sampling method was used to select individuals aged 50 and above as study subjects in an urban district of Nanjing, Jiangsu Province, East China. Firstly, 10 communities from two subdistricts (township-level regions) were randomly selected in Qixia District, Nanjing. Subsequently, residents were selected based on gender and age stratification, with an equal proportion of men and women, and a ratio of 2:2:1 for age groups 50–59, 60–69 and ≥ 70 years old. Eligible subjects were required to have resided in the local community for 5 years or more, voluntarily participate in this study, and be able to complete tests and answer questions independently. Individuals with stroke, dementia, and severe mental illness were excluded. A total of 1,114 subjects were selected for this study, and 1,045 subjects were finally included after processing outliers and missing data. All subjects provided informed consent.

### 2.2 KOA diagnosis

The diagnostic criteria for KOA released by the Chinese Medical Association in 2018 were used (21), including the following parameters: (1) Recurrent knee pain experienced within the past month; (2) X-ray examination (conducted in a standing or weight-bearing position) revealing joint space narrowing, subchondral bone sclerosis and/or cystic degeneration, as well as the presence of osteophytes at the joint edge; (3) Age ≥ 50 years old; (4) Morning stiffness lasting ≤30 min; (5) The presence of bone friction sound or sensation during activities. KOA diagnosis is established when both the first condition and any other two conditions are simultaneously met. Throughout this investigation, general practitioners first diagnosed KOA based on symptomatic manifestations such as pain, morning stiffness, and bone friction sound or sensation. In cases where a diagnosis could not be solely confirmed by symptoms, bilateral or unilateral knee X-ray examinations were assist in the diagnosis.

### 2.3 Risk factors

In this study, we screened 14 potential risk factors associated with KOA based on literature reports and clinician recommendations. Specifically, gender, age, weight, and body mass index (BMI) have been extensively validated as associated with KOA (22, 23) and were included to provide new data. Thigh circumference (TC) and calf circumference (CC) were selected based on clinical observations by local doctors, despite limited evidence linking them to KOA. The remaining eight factors are commonly reported in KOA epidemiological surveys, but no consensus has been reached regarding their associations with KOA (22–25); these were chosen to supplement the evidence in Nanjing. The 14 factors were categorized into three types. The first type refers to personal characteristics and lifestyle factors, including gender, age, marriage, education, smoking, and drinking. The second type comprises factors related to obesity and lower limb morphology, such as weight, BMI, TC, CC and flatfoot. The third type involves factors associated with lower limb strength and balance, including 30-s chair stand (30-s CS), single-leg stand (SLS), and timed up-and-go (TUG) tests.

### 2.4 Data collection

Data on these factors were collected through a self-designed questionnaire and standardized on-site tests. Some of the testing details were as follows. BMI data were calculated using the formula weight (kg) / height (m)^2^. According to the Chinese BMI evaluation criteria, BMI < 24 indicated that the subject was not overweight or obese, 24 ≤ BMI < 28 indicated overweight, and BMI ≥ 28 indicated obesity (26). For the TC and CC tests, we measured the thickest part of the thighs and calves separately, and calculated the averages of both legs. For the 30-s CS test, we recorded the number of repetitions of the subjects standing up and sitting down within 30 s. For the SLS test, subjects were asked to stand on one leg with their eyes closed, and we recorded the time until subjects open their eyes or move their supporting feet. For the TUG test, subjects were asked to stand up from an armchair, walk 3 meters forward, turn around at a marked line, walk back to the chair, turn around again, and sit down. Then we recorded the time taken to complete this sequence. Both the SLS and TUG tests were conducted twice, and the optimal values were used as the final data.

### 2.5 Statistical analysis

Epidata 3.1 software was used for data entry, while SPSS 21.0 software was utilized for single-factor or multiple-factor analysis. The dependent variable was the presence or absence of KOA, denoted by “yes” or “no.” The independent variables consisted of 14 potential KOA risk factors divided into 3 types. The normality of continuous variables was confirmed using the Kolmogorov-Smirnova test. Continuous data were presented as means ± standard deviations, and intergroup differences were assessed through independent-samples *t*-tests. Discrete data were presented as frequencies and composition ratios, with intergroup differences analyzed using Chi-square (*χ*^2^) tests. The significance level was set at 0.05. Subsequently, multiple logistic regression was used to establish risk factor models for KOA. Three models were developed by sequentially incorporating three types of risk factors using the stepwise entry method. Mode 1 was established based on personal characteristics and lifestyle factors. Model 2 expanded on Mode 1 by including obesity and lower limb morphology factors. Model 3 further incorporated factors related to lower limb strength and balance on the basis of Model 2. The fitting degree of these models was evaluated using the Hosmer-Lemeshow tests. Odds Ratios (ORs) and 95% Confidence Intervals (CIs) were used to quantitatively describe the association between risk factors and KOA.

## 3 Results

### 3.1 Descriptive characteristics and single-factor analysis results

This study included 1,045 subjects, among whom 247 suffered from KOA, resulting in a prevalence of 23.64%. The Kolmogorov-Smirnova test indicated that all continuous data followed a normal distribution. Single-factor analysis revealed that gender, age, smoking, drinking, weight, BMI, TC, CC, flatfoot, 30-s CS, SLS, and TUG were all associated with KOA. Specifically, the prevalence of KOA was 32.04% in women and 13.75% in men, with women exhibiting a 2.33 times higher prevalence than men. Among subjects aged 50–59, 60–60 and ≥ 70, the prevalence rates were 17.75, 25.93 and 31.00%, respectively, indicating an increasing trend with age groups. Smokers had a lower prevalence rate (14.23%) compared to non-smokers (26.43%), as did drinkers (16.33%) compared to non-drinkers (26.58%). Subjects with flatfoot exhibited a higher prevalence rate (30.88%) than those without flatfoot (18.49%). Notably, the prevalence of KOA among obese subjects was as high as 48.80%, which was 1.98 times higher than that among overweight subjects (24.59%) and 3.59 times higher than that among non-overweight or obese subjects (13.60%). Meanwhile, the mean weight, TC, CC, and TUG performance were higher among KOA subjects compared to non-KOA subjects, while the mean 30-s CS and SLS performance were lower among KOA subjects compared to non-KOA subjects. All of these differences were statistically significant (*p* < 0.05, Table 1). Furthermore, there were no significant differences in marriage and education between KOA subjects and non-KOA subjects (*p* > 0.05, Table 1).

**Table 1:** Descriptive characteristics and single factor comparisons of KOA risk factors.

Risk factors		Non-KOA	KOA	*χ*^2^/*t* value	*p* value
N		798 (76.36)^a^	247 (23.64)^a^		
Gender	Male	414 (86.25)^a^	66 (13.75)^a^	48.08^b^	< 0.01
	Female	384 (67.96)^a^	181 (32.04)^a^		
Age group	50–59	343 (82.25)^a^	74 (17.75)^a^	15.28^b^	< 0.01
	60–69	317 (74.07)^a^	111 (25.93)^a^		
	≥ 70	138 (69.00)^a^	62 (31.00)^a^		
Marriage	Married	736 (76.99)^a^	220 (23.01)^a^	2.42^b^	0.12
	Unmarried/divorced/widowed	62 (69.66)^a^	27 (30.34)^a^		
Education	Primary school and below	161 (72.20)^a^	62 (27.80)^a^	4.50^b^	0.11
	Junior high school	400 (79.05)^a^	106 (20.95)^a^		
	High school and above	237 (75.00)^a^	79 (25.00)^a^		
Smorking	Yes	205 (85.77)^a^	34 (14.23)^a^	15.20^b^	< 0.01
	No	593 (73.57)^a^	213 (26.43)^a^		
Drinking	Yes	251 (83.67)^a^	49 (16.33)^a^	12.43^b^	< 0.01
	No	547 (73.42)^a^	198 (26.58)^a^		
Flatfoot	Yes	94 (69.12)^a^	42 (30.88)^a^	4.55^b^	< 0.05
	No	704 (81.51)^a^	205 (18.49)^a^		
BMI	Non-overweight or obesity	394 (86.40)^a^	62 (13.60)^a^	83.89^b^	< 0.01
	Overweight	319 (75.41)^a^	104 (24.59)^a^		
	Obesity	85 (51.20)^a^	81 (48.80)^a^		
Weight (kg)		65.69 ± 10.44 ^c^	69.25 ± 11.31^c^	−4.59^d^	< 0.01
TC (cm)		50.11 ± 4.06^c^	51.69 ± 4.61^c^	−5.21^d^	< 0.01
CC (cm)		34.78 ± 2.65^c^	35.69 ± 3.04^c^	−4.20^d^	< 0.01
30-s CS (count)		19.77 ± 5.36^c^	16.91 ± 5.40^c^	6.94^d^	< 0.01
SLS (s)		7.69 ± 7.31^c^	5.61 ± 5.30^c^	4.89^d^	< 0.01
TUG (s)		9.79 ± 1.75^c^	10.57 ± 2.00^c^	−5.51^d^	< 0.01

a The data outside and inside parentheses are the frequencie and composition ratios (%), respectively.

b *χ2 value.*

c mean ± standard deviation.

d *t value.*

### 3.2 Multiple-factor analysis results

We established three logistic regression models. The *p*-values of the Hosmer-Lemeshow tests for Model 1, Model 2, and Model 3 were 0.87, 0.57, and 0.10, respectively, indicating good calibration of the models. In this study, both Model 1 and Model 2 identified gender and age group as significant independent risk factors for KOA (*p* < 0.05, Tables 2, 3), with Model 2 showing higher effect sizes. The results of Model 2 indicated that women (OR: 5.34, 95% CI: 3.13 to 9.11) were more likely to suffer from KOA compared to men, and subjects aged 60–69 (OR: 1.83, 95% CI: 1.25 to 2.69) and those over 70 (OR: 2.87, 95% CI: 1.80 to 4.59) had a higher likelihood of experiencing KOA compared to those aged 50–59. Moreover, both Model 2 and Model 3 identified education, flatfoot, and BMI as independent risk factors for KOA (*p* < 0.05, Tables 3, 4), with Model 3 demonstrating higher effect sizes. The results of Model 3 suggested that subjects with high school education and above (OR: 2.22, 95% CI: 1.37 to 3.60) were more likely to suffer from KOA compared to those with primary school education and below, and subjects with flatfoot (OR: 1.74, 95% CI: 1.10 to 2.74) were more predisposed to KOA compared to those without flatfoot. Similarly, overweight subjects (OR: 1.91, 95% CI: 1.21 to 3.04) and obese subjects (OR: 4.63, 95% CI: 2.18 to 9.85) were at an increased risk of KOA compared to those who were not overweight or obese based on their BMI status. Additionally, Model 2 also considered weight as an independent risk factor for KOA (*p* < 0.05, Table 3), indicating that the risk of KOA increased by 4% for every 1 kg increase in weight (OR: 1.04, 95% CI: 1.01 to 1.08). Model 3 revealed that 30-s CS and SLS were independent risk factors for KOA (*p* < 0.05, Table 4). The risk of KOA decreased by 6% for every 1 repetition increase in 30-s CS (OR: 0.94, 95% CI: 0.91 to 0.97) and by 4% for every 1 s increase in SLS (OR: 0.96, 95% CI: 0.93 to 0.99).

**Table 2:** Binary logistic regression model of personal characteristics and lifestyle factors related to KOA (Model 1).

Risk factors		*β*-value	OR (95% CI)	*p* value
Gender	Male		1.00	
	Female	1.19	3.27 (2.12–5.05)	< 0.01
Age group	50–59		1.00	
	60–69	0.40	1.50 (1.05–2.14)	< 0.05
	≥ 70	0.75	2.11 (1.37–3.25)	< 0.01
Marriage	Married		1.00	
	Unmarried/divorced/widowed	0.08	1.08 (0.65–1.80)	0.76
Education	Primary school and below		1.00	
	Junior high school	0.18	1.19 (0.80–1.78)	0.39
	High school and above	0.36	1.44 (0.94–2.21)	0.10
Smorking	No		1.00	
	Yes	0.09	1.09 (0.66–1.82)	0.73
Drinking	No		1.00	
	Yes	0.08	1.08 (0.70–1.68)	0.73

**Table 3:** Binary logistic regression model of personal characteristics, lifestyle, obesity, and lower limb morphology factors related to KOA (Model 2).

Risk factors		*β*-value	OR (95% CI)	*p* value
Gender	Male		1.00	
	Female	1.67	5.34 (3.13–9.11)	< 0.01
Age group	50–59		1.00	
	60–69	0.61	1.83 (1.25–2.69)	< 0.01
	≥ 70	1.05	2.87 (1.80–4.59)	< 0.01
Marriage	Married		1.00	
	Unmarried/divorced/widowed	0.02	1.02 (0.58–1.78)	0.95
Education	Primary school and below		1.00	
	Junior high school	0.21	1.24 (0.80–1.91)	0.34
	High school and above	0.62	1.85 (1.16–2.96)	< 0.05
Smorking	No		1.00	
	Yes	−0.01	1.00 (0.58–1.71)	0.99
Drinking	No		1.00	
	Yes	0.06	1.06 (0.66–1.70)	0.81
Flatfoot	No		1.00	
	Yes	0.47	1.61 (1.02–2.52)	< 0.05
BMI	Non-overweight or obesity		1.00	
	Overweight	0.62	1.86 (1.18–2.94)	< 0.01
	Obesity	1.45	4.25 (2.04–8.87)	< 0.01
Weight	—	0.04	1.04 (1.01–1.08)	< 0.05
TC	—	−0.02	0.98 (0.92–1.03)	0.40
CC	—	−0.02	0.98 (0.90–1.08)	0.73

**Table 4:** Binary logistic regression model of the 14 potential risk factors related to KOA (Model 3).

Risk factors		*β*-value	OR (95% CI)	*p* value
Gender	Male		1.00	
	Female	1.43	4.16 (2.41–7.20)	< 0.01
Age group	50–59		1.00	
	60–69	0.21	1.24 (0.80–1.88)	0.35
	≥ 70	0.48	1.62 (0.95–2.76)	0.08
Marriage	Married		1.00	
	Unmarried/divorced/widowed	−0.09	0.91 (0.52–1.62)	0.76
Education	Primary school and below		1.00	
	Junior high school	0.29	1.34 (0.86–2.08)	0.19
	High school and above	0.80	2.22 (1.37–3.60)	< 0.01
Smorking	No		1.00	
	Yes	−0.17	0.84 (0.48–1.46)	0.54
Drinking	No		1.00	
	Yes	0.15	1.16 (0.72–1.88)	0.55
Flatfoot	No		1.00	
	Yes	0.55	1.74 (1.10–2.74)	< 0.05
BMI	Non-overweight or obesity		1.00	
	Overweight	0.65	1.91 (1.21–3.04)	< 0.01
	Obesity	1.53	4.63 (2.18–9.85)	< 0.01
Weight	—	0.03	1.03 (0.99–1.06)	0.17
TC	—	−0.01	0.99 (0.94–1.05)	0.81
CC	—	0.01	1.01 (0.92–1.10)	0.91
30-s CS		−0.06	0.94 (0.91–0.97)	< 0.01
SLS		−0.04	0.96 (0.93–0.99)	< 0.05
TUG		0.10	1.10 (0.99–1.23)	0.07

## 4 Discussion

In this cross-sectional survey targeting middle-aged and older adults in Nanjing, China, we observed a KOA prevalence rate of 23.64%. This figure is lower than the prevalence rates in the United States (37.4%) and Japan (26.1%) among those aged 60 and older (10, 11), but higher than the rates found in England (17.4% for those over 50), Bangladesh (14.8% for those over 58), and Germany (12.3% for those over 60) (12, 27, 28). However, considering the influence of factors such as diagnostic methods for KOA, geographical location, ethnicity, and age range, the comparability of these figures requires further validation. Nevertheless, the high prevalence of KOA globally is undisputed. Furthermore, in China, the 23.64% prevalence is slightly higher than the estimate of 21.51% reported by Sun et al. after summarizing findings from 21 studies and significantly surpasses the estimate of 8.1% reported by Tang et al. (13, 14). Upon further analysis, we noted that both our study and the aforementioned 21 studies collected data through direct measurements, while the study of Tang et al. collected data through face-to-face household interviews and identified KOA patients by inquiring whether subjects had received a doctor’s diagnosis of KOA. Consequently, the estimate of 8.1% appears relatively low, and the prevalence rates of 23.64 and 21.51% may better reflect the actual prevalence, suggesting a serious challenge posed by KOA among the middle-aged and older adult population in Nanjing.

Subsequently, we investigated the risk factors for KOA and identified gender, age, education, flatfoot, weight, BMI, 30-s CS, and SLS as independent contributors. Gender and age emerged as the primary risk factors. Numerous studies, including ours, have consistently reported a higher prevalence of KOA among women compared to men (8, 10–12, 15, 29). This gender difference may be attributed to declining estrogen levels in perimenopausal women, which can diminish the metabolic capacity of joint cartilage and contribute to the onset of KOA. Additionally, women’s daily activities, such as squatting for defecation and participating in household chores, often entail repetitive stress on their knees, potentially accelerating knee joint wear and the onset of KOA. Furthermore, studies have consistently indicated an increasing prevalence of KOA with advancing age (13, 14, 29). This age-related trend was also observed in the univariate analysis, Model 1, and Model 2 of our study but not in Model 3. In contrast to Model 2, Model 3 incorporated three factors reflecting lower limb strength and balance function: 30-s CS, SLS, and TUG. We speculate that these three factors serve as intermediate variables linking age and KOA. With increasing age, the prevalence of KOA may rise due to declining muscle strength and deteriorating balance associated with aging, which may lead to knee joint wear and the development of KOA (30, 31).

After adjusting for other factors, education emerged as an independent risk factor for KOA. In both Model 2 and Model 3 of our study, we observed that the prevalence of KOA was 1.85 times and 2.22 times higher, respectively, in individuals with a high school education and above, compared to those with primary school education and below. However, some studies have reported contrasting results, suggesting a higher prevalence of KOA in individuals with lower education (20, 32). This discrepancy might be attributed to the association between KOA and occupation. Studies by Hulshof et al. and Zhou et al. suggested that individuals with lower education were more likely to engage in repetitive tasks such as kneeling, squatting, carrying heavy objects, and climbing stairs, which increased the risk of KOA (33, 34). Unfortunately, our study did not collect data related to occupational factors or other confounding factors, making it difficult to explain why opposite results were observed.

Flatfoot is recognized as an independent risk factor for KOA. Studies by Gross et al. and Lijima et al. have indicated that flatfoot is correlated with knee pain, knee cartilage damage, and can exacerbate disability in KOA patients (35, 36). Additionally, both Model 2 and Model 3 of our study reported that individuls with flatfoot had a 1.61 and 1.74 times greater risk of developing KOA, respectively, compared to those without flatfoot. The association between flatfoot and KOA can be explained through mechanical stress. During weight-bearing activities, the posture and movement of the feet and knees form a closed kinematic chain, working together to support weight and absorb impact. Flatfoot is characterized by weak arch support and limited ability to absorb impacts, inevitably leading to increased mechanical stress on the knee (37, 38). This stress may cause damage to the cartilage and soft tissue of the knee joint, thereby increasing the risk of KOA.

As is well-known, weight and BMI serve as significant risk factors for KOA (39, 40). This study revealed a notable association that with each additional 1 kg of weight, there was a 4% increase in the prevalence of KOA. Moreover, the study indicated that the prevalence of KOA among overweight individuals was 1.91 times higher compared to non-overweight and obese individuals, and the prevalence of KOA among obese individuals was 4.63 times higher in comparison. The mechanism by which weight and BMI impact KOA can be explained from two perspectives. Firstly, from a mechanical load perspective, the knee joint bears the greatest weight in the human body. As weight and BMI increase, the load on the knee joint escalates, heightening the risk of cartilage degradation. Secondly, from a fat metabolism perspective, higher weight and BMI are associated with increased body fat content. Fat-related factors can trigger inflammatory reactions in the joints, activate proteinases, and accelerate the degeneration of joint cartilage. Simultaneously, fat metabolism may interfere with cholesterol reverse transcription in joint cartilage, leading to cholesterol accumulation, hypertrophy of cartilage cells, cartilage ossification, and other factors that can trigger or exacerbate KOA (41–43).

The 30s-CS and SLS tests are not only widely used to objectively evaluate physical function in KOA patients, but are also considered as risk factors for KOA. This study reported that for every additional repetition in 30s-CS test and one-second increase in SLS test, the risk of KOA decreased by 6 and 4%, respectively. The 30s-CS reflects lower limb muscle strength, while SLS indicates lower limb static balance ability, and their association with KOA can be well explained. Specifically, insufficient muscle strength may lead to knee joint instability, causing it to swing during activities, thereby accelerating joint degeneration and contributing to KOA. Moreover, the correlation between muscle strength and KOA is influenced by gender, with women having less strength being more likely to suffer from KOA (44). The possible reason is that women have lower strength capacity and are closer to the risk threshold of KOA (45).

In this study, marriage, smoking, drinking, TC, CC, and TUG were not considered as independent risk factors for KOA. Regarding marriage, evidence suggests that it may be a risk factor for KOA, as the prevalence of KOA in married, divorced and widowed individuals was significantly higher than that in unmarried individuals (32, 46). However, our study did not find a significant correlation between marriage and KOA, which may be attributed to the relatively small sample size of unmarried, divorced, and widowed individuals, accounting for only 8.52% of the total. This sample size might not be sufficient to yield statistically significant results. Regarding smoking and drinking, the univariate analysis of our study suggested a notably lower prevalence of KOA among smokers or drinkers compared to non-smokers or non-drinkers, respectively, implying that smoking and drinking might act as protective factors for KOA. However, after adjusting for gender, age group, marriage, and education, no significant association was found between smoking or drinking and KOA, indicating the initial conclusion drawn from the univariate analysis was inaccurate. To investigate the reasons for this discrepancy, we further compared the differences in smoking and drinking among subjects of different genders, age groups, marriages, and educational levels using Chi-square tests. The results revealed substantial gender disparities in smoking (*χ*^2^ = 315.70, *p* < 0.01) and drinking (*χ*^2^ = 304.62, *p* < 0.01), with males exhibiting significantly higher rates than females. The erroneous conclusion likely stemmed from the interference of the gender factor. Regarding TC and CC, they to some extent reflect lower limb muscle mass and cross-sectional area, both of which have been demonstrated to be related to KOA (47, 48). Consequently, we attempted to explore the correlation between TC or CC and KOA, but unfortunately, we did not obtain statistically significant results. As for the TUG test, it has been widely utilized to evaluate the dynamic balance ability of KOA patients (49). The univariate analysis of our study also reported a significant association between TUG performance and KOA. However, the multivariate analysis did not confirm this association, as evidenced by a *p*-value of 0.07. We speculate that expanding the geographical and age distribution of the samples might influence this outcome, necessitating further validation.

## 5 Limitations

This study has several limitations. Firstly, all subjects were sourced from urban communities rather than rural areas, selected for the convenience of receiving KOA diagnosis in community hospitals, potentially introducing selection bias. It is recommended to broaden the sample coverage in future studies to ensure greater representativeness. Secondly, our study was a cross-sectional survey, which may reveal correlations between indicators and KOA, but cannot assess direct causality. Further cohort studies in Nanjing are necessary to address this limitation and provide more robust evidence.

## 6 Conclusion

The prevalence of KOA is remarkable in Nanjing city, indicating the urgency of developing and implementing targeted measures. This study demonstrates that factors such as women, older age, higher education, flatfoot, increased weight, higher BMI, as well as poor performance in 30s-CS and SLS tests, all contribute to the risk of KOA. These findings help identify vulnerable groups for KOA and are instrumental in the development of prevention and control measures for the condition.

## Data Availability

The datasets presented in this study can be found in online repositories. The names of the repository/repositories and accession number(s) can be found at: Figshare Dryad Digital Repository (https://doi.org/10.6084/m9.figshare.25679568).
